# Electrolyte Additives for Stable Zn Anodes

**DOI:** 10.1002/advs.202304549

**Published:** 2023-11-27

**Authors:** Shengchi Bai, Zhaodong Huang, Guojin Liang, Rui Yang, Di Liu, Wen Wen, Xu Jin, Chunyi Zhi, Xiaoqi Wang

**Affiliations:** ^1^ Research Institute of Petroleum Exploration & Development of China National Petroleum Corporation (RIPED) Beijing 100083 China; ^2^ Department of Materials Science and Engineering City University of Hong Kong 83 Tat Chee Avenue Kowloon Hong Kong SAR China

**Keywords:** aqueous Zn‐ion batteries, electrolytes, electrolyte additives, Zn anodes

## Abstract

Zn‐ion batteries are regarded as the most promising batteries for next‐generation, large‐scale energy storage because of their low cost, high safety, and eco‐friendly nature. The use of aqueous electrolytes results in poor reversibility and leads to many challenges related to the Zn anode. Electrolyte additives can effectively address many such challenges, including dendrite growth and corrosion. This review provides a comprehensive introduction to the major challenges in and current strategies used for Zn anode protection. In particular, an in‐depth and fundamental understanding is provided of the various functions of electrolyte additives, including electrostatic shielding, adsorption, in situ solid electrolyte interphase formation, enhancing water stability, and surface texture regulation. Potential future research directions for electrolyte additives used in aqueous Zn‐ion batteries are also discussed.

## Graphical abstract

This review discusses the challenges of Zn anodes and strategies for improving the stability of Zn anodes. Various electrolyte additives and their functions, including, in situ solid electrolyte interphase formation, enhancing water stability, and surface texture regulation, are thoroughly analyzed. Finally, the challenges and prospects for research on Zn anodes and their practical applications are discussed.

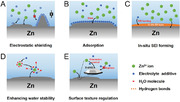

## 1 Introduction

Increases in fossil fuel consumption and the consequent environmental problems have resulted in renewable energy attracting considerable research interest globally. Solar energy and wind energy are among the most abundant energy sources; however, their intermittent nature makes it challenging to incorporate them into an electric grid. Developing electrochemical energy storage (EES) systems is key to constructing a smart grid that can efficiently regulate peak loads and that is more reliable, stable, and efficient than a conventional electrical grid.^[^ ^1^, ^2^ ^]^ Because of their high gravimetric energy density, long cycle life, and low weight, Li‐ion batteries (LIBs) have become the technology of choice for EES systems.^[^ ^3^, ^4^, ^5^ ^]^ However, the limited availability of, high cost of, and safety and pollution problems associated with lithium resources are considerably impeding the application of LIBs in large‐scale electrical energy storage systems. Thus, alternative rechargeable batteries with comparable energy density and superior safety to LIBs must be developed using abundant materials.^[^ ^6^, ^7^, ^8^ ^]^

Aqueous rechargeable metal‐ion batteries are inherently safe, environmentally benign, and economical, and have high ionic conductivity; therefore, these batteries are promising alternatives to LIBs.^[^ ^9^, ^10^, ^11^, ^12^ ^]^ Various aqueous metal‐ion batteries—including those using Li^+^,^[^ ^13^ ^]^ naturally abundant alkali metal ions (Na^+^ and K^+^),^[^ ^14^, ^15^, ^16^ ^]^ and multivalent charge carriers (Zn^2+^, Mg^2+^, Ca^2+^, and Al^3+^)^[^ ^17^, ^18^, ^19^, ^20^, ^21^, ^22^ ^]^—have been investigated. Of these batteries, aqueous Zn‐ion batteries (AZIBs) have received substantial attention and are considered the most promising batteries for future large‐scale EES systems because of the abundance of natural Zn reserves (≈300 times more abundant than lithium) and AZIBs' high specific capacitance (820 mAh g^−1^ and 5855 mAh cm^−3^), low redox potential (0.763 V vs standard hydrogen electrode), environmental nontoxicity, and low assembly requirements.^[^ ^23^, ^24^, ^25^, ^26^, ^27^ ^]^ AZIBs containing alkaline electrolytes—such as Zn–MnO_2_, Zn–metal, and Zn–air—have been investigated since the 1860s, and some have been commercialized. Rechargeable aqueous Zn–MnO_2_ batteries containing mild electrolytes have been reported.^[^ ^28^, ^29^, ^30^ ^]^ For example, Kang et al. reported a long‐cycle‐life AZIB that contains an α‐MnO_2_ cathode and 1 m ZnSO_4_ as the mild electrolyte.^[^ ^31^ ^]^ The substitution of an alkaline electrolyte with a near‐neutral electrolyte can reduce the severity of corrosion and the formation of insulation by‐products, enhancing the reversibility and cycle life of the AZIB.

Similar to LIBs, AZIBs are composed of a cathode, an anode, separators, and an electrolyte. In AZIBs, Zn^2+^ migrates between the cathode and the Zn anode during the charging–discharging process. However, many challenges—including cathode dissolution, Zn dendrite formation, interfacial side reactions of the Zn anode, and the low chemical stability and electrochemical stability of the electrolyte—must be addressed before AZIBs can be employed in practical applications.^[^ ^32^, ^33^ ^]^ Considerable efforts have been made to construct and optimize durable cathode materials with high capacity, including manganese oxides, vanadium oxides, and organic materials.^[^ ^34^, ^35^, ^36^ ^]^

Research on Zn anodes is critical for enhancing the cycling performance of AZIBs, which suffer from dendrite formation and side reactions, including hydrogen evolution reaction (HER) and corrosion, and many researchers have provided in‐depth reviews of such research. Yang et al. reviewed the fundamental aspects of dendrite formation on Zn anodes and strategies for suppressing such formation.^[^ ^37^ ^]^ Yuan et al. discussed the effectiveness of various methods for regulating the Zn‐electrolyte interphase in AZIBs.^[^ ^38^ ^]^ Geng et al. summarized the functions and mechanisms of different electrolyte additives and the structural design of Zn anodes.^[^ ^39^ ^]^ Many strategies have been reported to improve the performance of Zn anode, such as surface coating, structural optimization, electrolyte modification, and separator design. The surface coating can regulate interfacial electrochemical properties and processes, but factors including thickness, mechanical strength, and interface bonding should be noted for high‐performance Zn anode. Structural optimization methods such as nanostructure optimization, composition design, and crystal engineering can provide a better interface and hinder side reactions, but the cost problems still exist. The separator design method can regulate ion transport behavior, but the issues of uniformity and cost still need to be solved. Electrolyte modification, especially electrolyte additive engineering, is the simplest method for promoting Zn anode electrochemical performance, which can directly affect interfacial chemistry and dendrite‐free Zn deposition. Numerous electrolyte additives have been reported, such as succinonitrile,^[^ ^40^ ^]^ trimethylethyl ammonium trifluoromethanesulfonate,^[^ ^41^ ^]^ CeCl_3_, ^[^ ^42^ ^]^ and others. Besides, developing in situ techniques, especially transmission electron microscopy with closed liquid cells, also helps in the mechanism study of novel electrolyte additives. This opens up the possibility of directly observing and analyzing liquid specimens without freezing or drying processes.^[^ ^43^, ^44^ ^]^

In this review, we discuss the current challenges involved in the application of Zn anodes and strategies for Zn anode protection. Furthermore, the functions of various electrolyte additives—such as electrostatic shielding, adsorption, in situ solid electrolyte interphase (SEI) formation, enhancing water stability and surface texture regulation—are analyzed in detail. Finally, given that the development of electrolyte additives for Zn anodes remains in the incipient stage, the challenges involved in and prospects for academic research on Zn anodes and their practical applications are described. This review is aimed at providing deeper insight into the exploration and optimization of electrolyte additives and enhancing the practical application of AZIBs in grid‐scale EES systems.

## 2 Current Challenges in and Strategies for Zn Anode Protection

In AZIBs, a Zn anode is used in a mild electrolyte, such as ZnSO_4_ solution. Zn is abundant, cheap, and environmentally friendly in aqueous electrolyte; however, Zn anodes have poor stability. This section introduces the main problems associated with the use of Zn anodes and the corresponding strategies used to overcome these problems.

### 2.1 Challenges Involved in Using Zn Anodes

**Figure 1:**
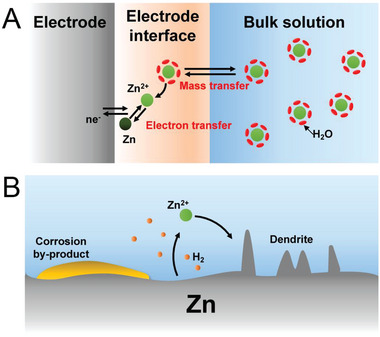
Schematics of A) the plating–stripping process of the Zn anode in AZIBs (not to scale) and B) the problems associated with the use of a Zn anode in a mild electrolyte, including dendrite formation, H_2_ evolution, and electrode corrosion.

In widely used mild electrolytes, the Zn anode reaction is a reversible electrochemical reaction between elemental Zn and Zn^2+^ that occurs during the discharging–charging process (**Figure** **1**A). During the electrical plating process, the formation of Zn at the anode–electrolyte interface involves four successive phenomena: Zn^2+^ mass transfer, desolvation, electrochemical reduction, and electrocrystallization growth.^[^ ^45^, ^46^, ^47^ ^]^ Theoretical and experimental results indicate that the deposition morphology is affected by many factors, such as the adopted substrate, current density, applied voltage, ion concentration, and electrolyte. Zn electrodes have higher reversibility in mild electrolyte systems than in alkaline electrolyte systems; however, the coulombic efficiency (CE) achieved in the plating–stripping process is poor because of the formation of Zn dendrites and the occurrence of side reactions, such as the HER and electrode corrosion (Figure 1B).^[^ ^48^, ^49^, ^50^ ^]^

The plating–stripping process of Zn plays a vital role during the discharging–charging process, and Zn deposition that is more uniform and compact leads to higher cycling performance and CE of AZIBs. However, Zn tends to form in ramified morphologies, especially dendrite‐like morphologies, during the electrodeposition process. The main reasons for dendrite growth are an uneven electric field distribution and uncontrollable 2D diffusion of Zn^2+^ on the anode surface. The Zn^2+^ plating–stripping process is dominated by liquid‐phase mass transfer, surface polarization, and 2D diffusion; thus, dendrites grow on the surface Zn anode and are then consolidated because of the “tip effect,” which causes Zn^2+^ to be deposited at the dendrite bulges on minimal surfaces. This “tip effect” intensifies the unevenness of the electric field distribution and dendrite growth, resulting in inhomogeneous Zn deposition.^[^ ^51^, ^52^, ^53^ ^]^ The formation of sharp and needle‐like Zn dendrites may lead to the piercing of separators, thereby causing a short circuit and battery failure. Moreover, the formation of Zn dendrites with a loose and porous structure can cause a decrease in the quantity of Zn participating in the electrochemical process because of the fracturing and disconnection of dendrites forming “dead” Zn.^[^ ^54^, ^55^, ^56^ ^]^

In addition to dendrite formation, side reactions caused by the aqueous solution, including the HER and corrosion, affect the performance of an AZIB. Zn anodes are thermodynamically unstable in weakly acidic electrolytes because of the high H^+^ activity of these electrolytes. Although the HER has a large overpotential, the electrolyte is still consumed in continuous reactions that proceed at a low rate, which leads to low CE and a shortened cycle life.^[^ ^57^, ^58^ ^]^ Besides, the presence of a solvation shell of zinc ion forming (Zn(OH_2_)_6_)^2+^ in mildly acidic conditions leads to sluggish kinetics, and the desolvation process heightens interfacial polarization, resulting in large charge transfer impedance and promotes HER.^[^ ^59^, ^60^ ^]^ Furthermore, the HER is accompanied by corrosion of the Zn anode, which is mainly attributed to electrochemical corrosion through H_2_ evolution in the mild electrolyte. In the electrochemical corrosion reaction, impurities function as a cathode, and numerous corrosion cells thus form. This corrosion process consumes H^+^, changes the local pH, and generates loose and insulative by‐products on the Zn anode. In contrast to the dense and uniform SEI layer on LIBs, the aforementioned loose and porous interphase layer cannot protect the fresh Zn anode from corrosion, which leads to continuous anode consumption and worsening electrical contact.^[^ ^61^, ^62^, ^63^, ^64^ ^]^

Moreover, the dendrite growth and side reactions have significant interactions. The dendrites lead to an increment of Zn metal anode surface area, accelerating HER and corrosion. Side reactions result in uneven surfaces, leading to nonuniform Zn deposition and dendrite growth.^[^ ^59^, ^62^ ^]^

### 2.2 Current Strategies for Zn Anode Protection

**Figure 2:**
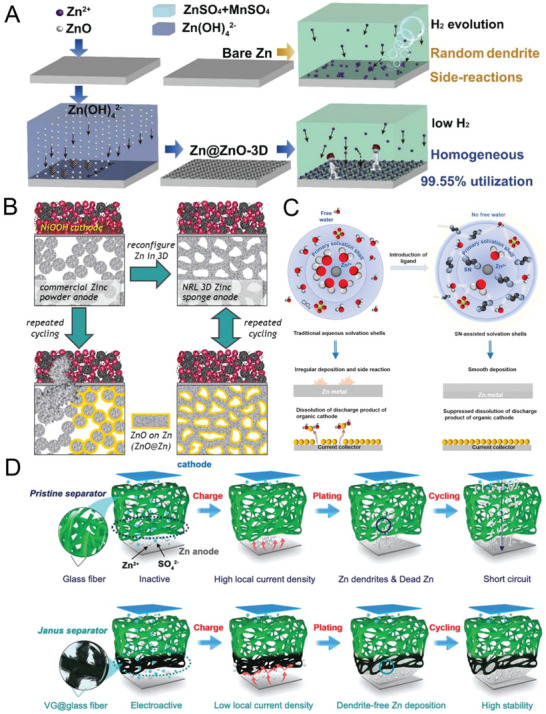
Current reported strategies for obtaining dendrite‐free and stable Zn anodes: A) surface‐coating‐layer modification. Reproduced with permission.^[^ ^65^ ^]^ Copyright 2020, Royal Society of Chemistry. B) internal structural optimization. Reproduced with permission.^[^ ^66^ ^]^ Copyright 2017, American Association for the Advancement of Science. C) electrolyte modification. Reproduced with permission.^[^ ^40^ ^]^ Copyright 2020, Elsevier and D) separator design. Reproduced with permission.^[^ ^67^ ^]^ Copyright 2020, John Wiely and Sons.

Nucleation regulation and side reaction suppression are two vital strategies used to obtain a dendrite‐free and stable Zn anode and thus achieve a high‐performance AZIB. Many strategies have been developed for alleviating or solving dendrite formation and side reactions; these strategies include surface‐coating‐layer modification of the planar Zn electrode, internal structural optimization of the Zn bulk electrode, electrolyte modification, and multifunctional separator design. The mechanism diagrams of typical research are shown in **Figure** **2**.^[^ ^40^, ^65^, ^66^, ^67^ ^]^

#### 2.2.1 Surface‐Coating‐Layer Modification of Zn Anodes

Zn anodes, such as Zn plates and powders, have been commercialized and are abundantly available on the market; thus, Zn anodes have become the anodes most widely used in AZIBs. Zn plates are most commonly used; however, planar anodes with a limited specific area can cause nucleation, a low Zn utilization ratio, and the electric field distribution to be inhomogeneous. The surface‐coating layer on these anodes can be modified to form an artificial interface layer that is similar to the SEI layer in LIBs. This strategy can regulate the electrochemical environment of the surface of the Zn anode, and the produced artificial interface layer can act as a physical layer protecting the Zn anode.

**Figure 3:**
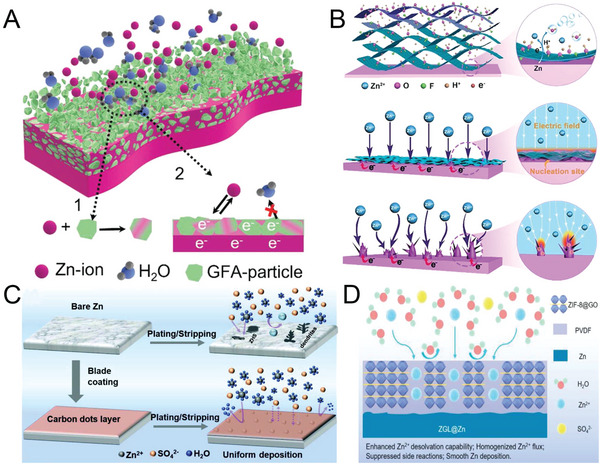
A) Schematic of Zn‐ion transfer and the electron flow pathway during the Zn deposition process on gradient fluorinated alloy coating layer. Reproduced with permission.^[^ ^70^ ^]^ Copyright 2008，Royal Society of Chemistry. B) Illustration of a synchronously reducing and assembling MXene layer on the surface of a Zn foil. Reproduced with permission.^[^ ^71^ ^]^ Copyright 2020, John Wiely and Sons. C) Schematic of the stabilization effect of carbon dots on a Zn anode. Reproduced with permission.^[^ ^77^ ^]^ Copyright 2022, John Wiely and Son and D) Illustration of a bare Zn electrode and an electrode containing ZIF‐8, GO@Zn, and polyvinylidene difluoride during the Zn^2+^ deposition process. Reproduced with permission.^[^ ^83^ ^]^ Copyright 2022, Elsevier.

Many materials such as metal oxide (sulfide) nanomaterials,^[^ ^65^, ^68^, ^69^, ^70^, ^71^ ^]^ inorganic acid salts,^[^ ^72^, ^73^ ^]^ metal–organic framework based materials, covalent organic framework based materials,^[^ ^74^, ^75^, ^76^ ^]^ carbon materials,^[^ ^77^, ^78^ ^]^ polymer materials,^[^ ^79^, ^80^, ^81^ ^]^ and organic–inorganic hybrid materials^[^ ^82^, ^83^ ^]^ have been used for modifying the surface of Zn anodes. In one study, a simple in situ‐constructed, 3D coating layer was designed; this layer was composed of spatial gradient fluorinated alloy nanoparticles with ZnF_2_ on the outside and Cu–Zn alloy on the inside.^[^ ^70^ ^]^ This protective layer caused the formation of dendrite‐free morphology through two mechanisms: 1) by storing plated Zn through the formation of Cu–Zn alloy inside Cu–Zn particles and 2) by filling the voids between the gradient fluorinated alloy particles, as shown in **Figure** **3**A. An ultrathin and uniform MXene layer on the surface of Zn anodes can reduce the Zn nucleation energy barrier and enhance the uniformity of the electric field through favorable charge redistribution (Figure 3B).^[^ ^71^ ^]^ He et al. engineered hydroxymethyl Zn phosphates (Zn(O_3_PCH_2_OH)) as an artificial functional layer for high‐rate durable AZIBs.^[^ ^84^ ^]^ The order hexagonal ion channels with a much smaller diameter provide effective physical interceptions for hydrated ions and polyanions. The targeted hydrogen‐bonding interactions between the abundant ─OH groups located in hexagonal ion channels and the water molecules in hydrated Zn^2+^ remarkably enhance the desolvation process, leading to ultrahigh‐rate endurability of up to 50 mA cm^−2^, with the overpotential being 36% lower than that of a bare Zn anode.

Carbon‐based materials have also been investigated as a coating layer for Zn anodes. In one study, functional carbon dots were adopted as a solid electrolyte protection layer to enhance the electrochemical stability of a Zn electrode.^[^ ^77^ ^]^ This zincophilic and inert layer induced uniform Zn deposition and inhibited dendrite growth because of the binding effect and homogenous electric field distribution. Moreover, the carbon dots layer alleviates Zn corrosion and lowers water reactivity due to electrostatic repulsion, as shown in Figure 3C. Gan et al. reported a multifunctional protective layer consisting of zeolitic imidazolate framework‐8 (ZIF‐8), decorated graphene oxide (GO), and polyvinylidene difluoride (Figure 3D).^[^ ^83^ ^]^ This protective layer isolates the Zn anode from the electrolyte to minimize side reactions, and the ZIF‐8 nanoparticles on GO homogenize the Zn^2+^ flux distribution and notably reduce the desolvation activation energy, thereby endowing the Zn anode with high electrochemical stability during consecutive whole cycles.

#### 2.2.2 Internal Structural Optimization of the Zn Bulk Electrode

In addition to surface modification, internal structural optimization is a promising strategy for producing high‐performance Zn electrodes. This strategy involves nanostructure optimization, composition design, and crystal engineering. Nanostructure optimization can enhance cycling performance by producing exposed surfaces, accelerating ion transportation, and reducing volume expansion during battery cycling. Composition design, especially alloying, can effectively promote cycling performance by protecting Zn anodes from corrosion and providing better interfaces for nucleation. Crystal orientation can considerably affect anode performance. The production of epitaxial zinc anodes by using graphene or other materials can effectively solve the problem of dendrite formation. The variation produced in the interphase lattice mismatch during such production can ensure that the deposited Zn has a locked crystal orientation.^[^ ^54^ ^]^

**Figure 4:**
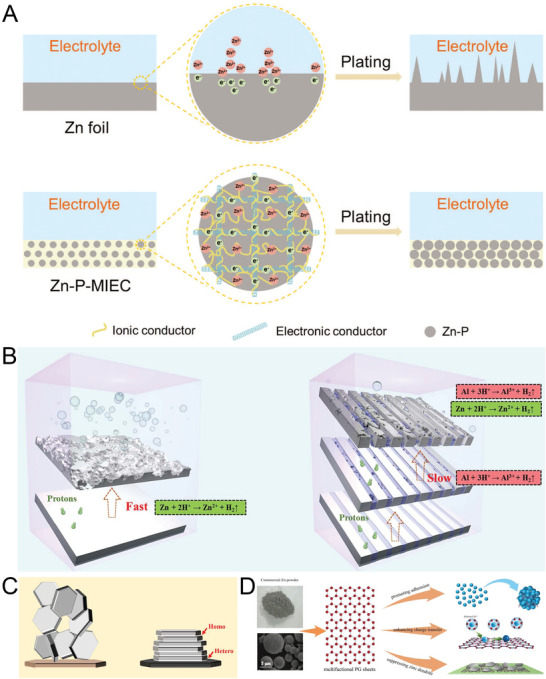
A) Schematic of the morphology evolution of Zn powder containing mixed ionic–electronic conductors during Zn stripping–plating cycling. Reproduced with permission.^[^ ^87^ ^]^ Copyright 2022, John Wiely and Son. B) Schematic diagram of corrosion process of Zn and Zn‐Al alloy electrodes. Reproduced with permission.^[^ ^92^ ^]^ Copyright 2023, Elsevier. C) Schematic of the design principle of epitaxial metal electrodeposition,Reproduced with permission.^[^ ^7^ ^]^ Copyright 2019, American Association for the Advancement of Science. and D) use of multifunctional pristine graphene for constructing an anode made of zinc powder. Reproduced with permission.^[^ ^93^ ^]^ Copyright 2022, Elsevier.

Planar Zn foil is conventionally utilized as a Zn anode. However, Zn foil is often excessively thick (20–300 µm) and thus leads to AZIBs with low energy density. The use of Zn foil can result in dendrite formation because of the nonuniform interfacial electric field and zinc deposition induced by the planar foil structure. Furthermore, the pulverization of an entire piece of Zn foil during continuous cycling can result in poor electric contact and battery failure. Thus, a promising solution for enhancing the performance of AZIBs involves replacing the planar Zn electrode through nanostructure engineering to enhance the anode's performance.^[^ ^85^, ^86^ ^]^ Parker et al. reported a 3D Zn sponge anode with high cycling durability; the porous structure of this anode can suppress the formation of separator‐piercing dendrites.^[^ ^66^ ^]^ Constructing a composite 3D Zn structure by using skeletal material is a more common approach than is producing pure 3D Zn anodes. In one study, a mixed ionic–electronic conducting scaffold was introduced into Zn powder to fabricate anticorrosive, flexible, and dendrite‐free Zn anodes.^[^ ^87^ ^]^ The guidance of electron conduction resulted in a uniformly redistributed electric field; thus, the tip effect was avoided. Moreover, the uniform ion conduction outside the Zn powder ensured that a greater quantity of homogeneous Zn^2+^ quickly entered the electrode and produced uniform Zn deposition, as shown in **Figure** **4**A.

Composition design, such as alloying, is an effective method for optimizing the performance of Zn anodes.^[^ ^88^, ^89^, ^90^ ^]^ Korobov et al. systematically examined the effects of different Zn‐based alloys on cycling performance in alkaline solution.^[^ ^91^ ^]^ The alloying method should be modified in accordance with the electrolyte and chemical environment. Cai et al. investigated the corrosion of Cu–Zn composites in ZnSO_4_ aqueous electrolyte.^[^ ^61^ ^]^ These composites had higher stability than did bare Zn electrodes, which was attributed to the high chemical stability and less negative electrode potential of Cu; thus, Cu–Zn composite electrodes corroded at a considerably lower rate than did bare Zn electrodes (−0.964 V and 6.03 µA cm^−2^ for Cu–Zn versus −0.976 V and 37.15 µA cm^−2^ for bare Zn) in aqueous electrolyte. In addition to Cu, Al can be combined with Zn to yield Zn–Al alloy. The use of Zn–Al alloy anodes synthesized using a metallurgic approach in Zn–MnO_2_ batteries was previously investigated.^[^ ^92^ ^]^ The preferential but slow corrosion of the Al matrix protected Zn from acid corrosion and thus enhanced its corrosion resistance, as shown in Figure 4B.

Zn dendrite formation is mainly attributed to the existence of a nonuniform interfacial electric field; thus, regulating the orientation of Zn deposition is an effective method for avoiding the formation of vertically oriented Zn. Zheng et al. proposed the concept of reversible epitaxial electrodeposition, according to which graphene acts as a substrate with small lattice mismatch for a Zn anode and thus can control the Zn anode's epitaxial growth pattern (Figure 4C).^[^ ^7^ ^]^ A Zn anode can achieve high reversibility (CE > 99.7%) at moderate and high current densities. An electrochemically inactive graphene substrate has a similar atomic arrangement to Zn metal, and the lattice mismatch between graphene (0002) and Zn (0002) is ≈7%, which implies the formation of a semicoherent interface on the crystal plane of graphene. Thus, the heteroepitaxial nucleation and growth of the Zn anode are accelerated, which leads to homoepitaxial deposition. In one study, a Zn powder–pristine graphene anode with smooth deposition kinetics and dendrite‐free deposition morphology was produced.^[^ ^93^ ^]^ Pristine graphene can be integrated with loose Zn powder to fabricate electrodes and improve cycling performance by enhancing charge transfer and promoting the horizontal alignment of zinc deposition, as shown in Figure 4D.

#### 2.2.3 Electrolyte Modification

The electrolyte in an AZIB has a vital influence on the battery's performance, and its chemical composition directly affects the diffusion behavior and electrochemical reactions involving Zn^2+^. Two strategies, namely the use of an electrolyte additive and the use of a water‐deficient electrolyte, have been adopted to enhance the performance of Zn anodes.^[^ ^38^, ^40^, ^94^ ^]^ Soluble additives have been used to enhance the performance of cathode materials^[^ ^95^, ^96^, ^97^ ^]^ and Zn anodes. Electrolyte additives for Zn anodes can be classified into four types: ionic, inorganic, organic, and metal. For instance, the triple‐function electrolyte additive C_3_H_7_Na_2_O_6_P can regulate H^+^ concentration, reduce free‐water activity to inhibit H_2_ evolution, and lead to formation of a self‐healing SEI.^[^ ^98^ ^]^

**Figure 5:**
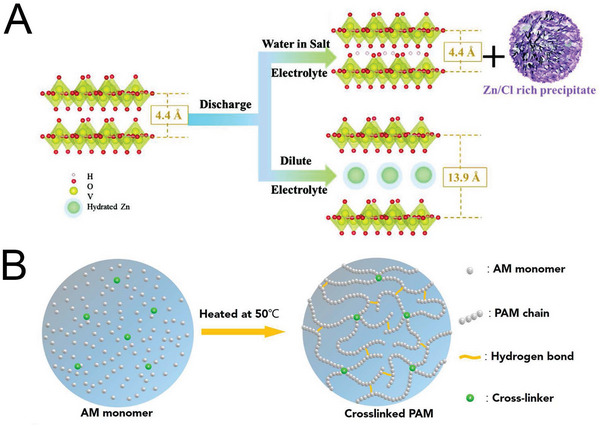
A) Schematic of the dynamic structural evolution of a V_2_O_5_ cathode in different electrolytes. Reproduced with permission.^[^ ^104^ ^]^ Copyright 2021, John Wiely and Son. B) Schematic diagram for the fabrication of the cross‐linked polyacrylamide ‐based electrolyte. Reproduced with permission.^[^ ^114^ ^]^ Copyright 2018, American Chemical Society.

Another useful strategy to enhance the performance of Zn anodes involves reducing the quantity of water in the electrolyte (e.g., by producing a water‐in‐salt electrolyte).^[^ ^99^, ^100^, ^101^ ^]^ In aqueous electrolytes, all reactions are related to the strong interactions between excessive water and Zn metal. The strong coordination bonds formed between water molecules and Zn^2+^ increase the desolvation energy of hydrated zinc ions, which is harmful to Zn deposition.^[^ ^29^ ^]^ Thus, reducing the water content to yield water‐deficient electrolytes (mainly water‐in‐salt electrolytes) is a promising method for enhancing the performance of Zn anodes. Water‐in‐salt electrolyte systems contain limited free water; this can suppress dendrite formation and widen the potential window for high energy density.^[^ ^102^, ^103^ ^]^ Tang et al. employed a high concentration of ZnCl_2_ water‐in‐salt electrolyte (30 m) to reduce the internal mechanical stress of the cathode and inhibit Zn dendrite deposition.^[^ ^104^ ^]^ A high‐voltage and low‐cost AZIB containing a zinc perchlorate water‐in‐salt electrolyte was previously reported.^[^ ^105^ ^]^ The free water in the electrolyte was found to be efficiently suppressed by the trapping of water molecules in the solvation sheath of Zn^2+^ cations, as shown in **Figure** **5**A.

The gel‐based quasi‐solid electrolyte is an ideal alternative to liquid electrolyte by further weakening the impact of water on the Zn anode. The stable polymer hydrogel retains a small amount of water, forming a gel‐based quasi‐solid electrolyte with flexibility, stability, mechanical strength, and high ionic conductivity. This gel electrolyte can also be a protective layer on the Zn anode for better cycling performance. Recently, many hydrophilic polymers have been studied for gel‐based quasi‐solid electrolytes, such as poly(ethylene oxide),^[^ ^106^, ^107^ ^]^ poly(vinyl alcohol),^[^ ^108^, ^109^ ^]^ poly(vinylidene fluoride),^[^ ^110^ ^]^ polyacrylamide,^[^ ^111^, ^112^ ^]^ and others.^[^ ^113^ ^]^ Li et al. developed a high‐performance, waterproof, tailorable, and stretchable yarn AZIB using a cross‐linked polyacrylamide electrolyte (Figure 5B).^[^ ^114^ ^]^ The gel electrolyte acts as an effective separator and an excellent ionic conductor, leading to a high‐performance battery with a specific capacity and volumetric energy density of 302.1 mAh g^−1^ and 53.8 mWh cm^−3^, respectively.

#### 2.2.4 Multifunctional Separator Design

The separator is a vital component of AZIBs; it averts short‐circuiting by preventing physical contact between the cathode and anode. Commonly used separators, such as glass fiber and filter paper, are inactive and cannot regulate Zn plating–stripping behavior. These traditional separators can lead to an ion concentration gradient near the zinc anode, which results in the growth of Zn dendrites. Thus, the morphological or chemical engineering of separators by using surface coating and functionalization strategies is a feasible approach for achieving high‐performance AZIBs.

**Figure 6:**
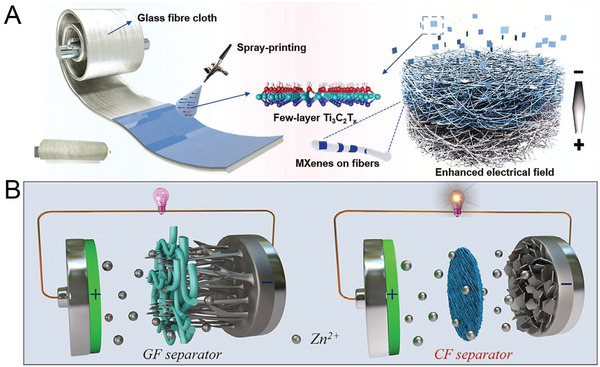
A) Schematic and photographs of the preparation and function of MXene–glass‐fiber Janus separators. Reproduced with permission.^[^ ^119^ ^]^ Copyright 2022, John Wiely and Son and illustration of the difference in zinc plating behavior with glass fiber and cellulose‐film separators. Reproduced with permission.^[^ ^122^ ^]^ Copyright 2022, Elsevier.

Several strategies have been proposed for modifying the separator in AZIBs; these strategies include the use of functional groups, the coating of the separator surface, the construction of hybrid architectures, the regulation of porosity, and the adoption of a bipolar membrane.^[^ ^67^, ^115^, ^116^, ^117^ ^]^ The strong interactions between functional groups or surface‐coating materials within separators and electrolyte ions directly control Zn^2+^ transportation and Zn deposition behaviors.^[^ ^67^, ^118^ ^]^ The Janus separator was designed by spraying a Ti_3_C_2_T*_x_* MXene layer on one side of a commercial separator; this resulted in the presence of abundant surface polar groups, high electrolyte wettability, and high ionic conductivity, as shown in **Figure** **6**A. Thus, the local current distribution was homogenized, and Zn nucleation kinetics were promoted.^[^ ^119^ ^]^

Regulating the porosity of the separator is also an effective method for enhancing anode performance because the diffusion of electrolyte ions mainly occurs through the pores of the separator rather than within the scaffold of the separator.^[^ ^120^, ^121^ ^]^ Compared with glass‐fiber separators, which have large and uneven pores, cellulose‐film separators made from cotton provide homogeneous and dense nanopores that promote Zn^2+^ transfer, homogenize the ion flux, and accelerate the Zn deposition kinetics at the electrode–electrolyte interface (Figure 6B).^[^ ^122^ ^]^ Bipolar membranes can increase the concentrations of H^+^ and OH^−^ at the cathode and Zn anode during the electrochemical process, which leads to the formation of a protective layer on the Zn anode.^[^ ^123^ ^]^

## 3 Electrolyte Additives for Zn Anodes

**Figure 7:**
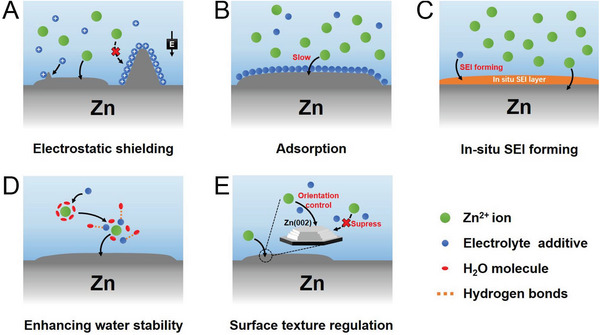
Modification mechanisms involving electrolyte additives in AZIBs: A) electrostatic shielding, B) adsorption, C) in situ SEI formation, D) enhancing water stability, and E) surface texture regulation.

The introduction of electrolyte additives to AZIB systems is the most convenient and economical method for enhancing the performance of Zn anode. Numerous electrolyte additives have been developed for high‐performance Zn anode, as listed in **Table** **1**. The mechanisms through which electrolyte additives exert effects in AZIBs are electrostatic shielding, adsorption, in situ SEI formation, enhancing water stability, surface texture regulation etc. (**Figure** **7**).

**Table 1:** Summary of electrolyte additives and their effects on Zn anode.

Mechanism	Electrolyte	Additives	Cell performance
Electrostatic shielding	2 m ZnSO_4_	CeCl_3_^[^ ^42^ ^]^	Zn/Zn symmetric cell: 2600 h at 2 mA cm^−2^
	1 m ZnSO_4_	NaSO_4_^[^ ^124^ ^]^	Zn/NaV_3_O_8_·1.5H_2_O battery: capacity retention of 82% after 1000 cycles at 4 A g^−1^
	2 m ZnSO_4_	Ammonium hydroxide^[^ ^125^ ^]^	Zn/Zn symmetric cell: 1500 h at 1 mA cm^−2^
	3 m ZnSO_4_	Y_2_(SO_4_)_3_^[^ ^126^ ^]^	Zn/Zn symmetric cell: 2080 h at 5 mA cm^−2^
	3 m Zn(CF_3_SO_3_)_2_ + 0.1 m Mn(CF_3_SO_3_)_2_	Et_2_O^[^ ^127^ ^]^	Zn/MnO_2_ battery: capacity retention of 97.7% after 4000 cycles at 5 A g^−1^
Adsorption	1 m ZnSO_4_	Polyethylene oxide^[^ ^128^ ^]^	Cu/Zn cell: 3000 h at 1 mA cm^−2^
	3 m ZnSO_4_	Perfluorooctanoic acid^[^ ^129^ ^]^	Zn/Zn symmetric cell: 2200 h at 1 mA cm^−2^
	2 m ZnSO_4_	Tetrabutylammonium sulfate^[^ ^130^ ^]^	3D Zn/3D Zn symmetric cell: 300 h at 2 mA cm^−2^
	1 m ZnSO_4_	Cetyltrimethyl ammonium bromide^[^ ^131^ ^]^	Zn/Zn symmetric cell: 2000 h at 2 mA cm^−2^
	1 m ZnSO_4_	Sodium 3‐mercapto‐1‐propanesulfonate^[^ ^132^ ^]^	Zn/Zn symmetric cell: 4500 h at 1 mA cm^−2^
	2 m ZnSO_4_	Ti_3_C_2_T_x_ MXene^[^ ^133^ ^]^	Zn/Zn symmetric cell: 1180 h at 2 mA cm^−2^
In‐situ SEI formation	2 m ZnSO_4_	Saccharin^[^ ^134^ ^]^	Zn/Zn symmetric cell: 550 h at 10 mA cm^−2^
	2 m ZnSO_4_	Compounding corrosion inhibitor^[^ ^135^ ^]^	Zn/Zn symmetric cell: 1100 h at 1 mA cm^−2^
	2 m Zn(OTF)_2_	ZnSO_4_/NaSO_4_^[^ ^136^ ^]^	Zn/Zn symmetric cell: 2000 h at 1 mA cm^−2^
Enhancing water stability	2 m ZnSO_4_	1,2‐dimethoxyethane^[^ ^137^ ^]^	Zn/V_2_O_5_ battery: capacity retention of 54.2% after 3000 cycles
	2 m ZnSO_4_	*N*, *N*‐dimethyl acetamide^[^ ^138^ ^]^	Zn/Zn symmetric cell: 4500 h at 1 mA cm^−2^
	2 m ZnSO_4_	*N*‐methyl‐2‐pyrrolidone^[^ ^139^ ^]^	Cu/Zn cell: 2000 h at 1 mA cm^−2^
	2 m ZnSO_4_	Methanol^[^ ^140^ ^]^	Zn/PANI battery: capacity retention of 89.3% after 2000 cycles at 5 A g^−1^ and −10 °C
Surface texture regulation	3 m ZnSO_4_	Zn(BF_4_)_2_^[^ ^141^ ^]^	Zn/(NH_4_)V_6_O_13_·H_2_O: no capacity degradation after 2000 cycles at 2 A g^−1^
	1 m ZnSO_4_	Sodium dodecyl sulfate^[^ ^142^ ^]^	Zn/Zn symmetric cell: capacity retention of 79% after 1000 cycles
	2 m ZnSO_4_	1,4‐dioxane^[^ ^143^ ^]^	Zn/(NH_4_)V_4_O_10_ battery: capacity retention of 95.4% after 1000 cycles at 5 A g^−1^

### 3.1 Electrostatic Shielding

**Figure 8:**
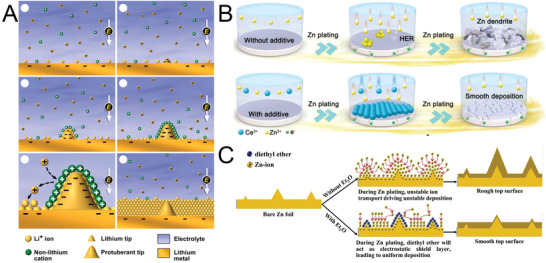
A) Li deposition under the self‐healing electrostatic shielding mechanism. Reproduced with permission.^[^ ^144^ ^]^ Copyright 2013, American Chemical Society. B) Schematic of the effect of CeCl_3_ additive on the Zn deposition process. Reproduced with permission.^[^ ^42^ ^]^ Copyright 2022, John Wiely and Son. C) Schematic of the morphological evolution of Zn anodes in mild aqueous electrolyte with and without diethyl ether additive during Zn stripping–plating cycling. Reproduced with permission.^[^ ^127^ ^]^ Copyright 2019, Elsevier.

Ding et al. reported the occurrence of electrostatic shielding in LIBs with Cs^+^ and Rb^+^ as additives.^[^ ^144^ ^]^ According to the Nernst equation, an additive cation has a lower effective reduction potential than does Li^+^ if the additive cation has lower chemical activity than does Li^+^ and is present in a low concentration. Additive cations with a lower reduction potential that that of Li^+^ accumulate in the vicinity of the initial growth tip of protuberances anode to form an electrostatic shield around this tip; thus, lithium deposition shifts to adjacent regions on the anode, and dendrite formation in LIBs is eliminated (**Figure** **8**A).

Electrostatic shielding is also adopted in aqueous electrolyte batteries to inhibit Zn dendrite formation. Hu et al. explored the use of cerium chloride as a low‐cost, effective, and nontoxic electrolyte additive.^[^ ^42^ ^]^ The introduction of Ce^3+^ as a competitive cation for Zn^2+^ results in the formation of a dynamic electrostatic shielding layer on the Zn metal anode, which forces Zn^2+^ to be deposited in adjacent flat regions, thereby leading to homogeneous and dendrite‐free Zn deposition. Zn–Zn symmetric cells with cerium chloride as an electrolyte additive achieved long cycling stability of 2600 h at 2 mA cm^−2^ and high CE of ≈99.7%, as shown in Figure 8B. Cations other than Ce^3+^ can act as an electrostatic shielding layer.^[^ ^124^ ^]^ In one study, a low‐cost, dual‐functional ammonium acetate additive was adopted to suppress dendrite formation and side reactions in an AZIB.^[^ ^145^ ^]^ NH_4_^+^, which binds to Zn anode with energy of considerably higher magnitude (−2.10 eV) than Zn^2+^ (−0.99 eV), can be preferentially adsorbed on the surface of Zn to form an electrostatic shielding layer that inhibits dendrite growth, even with trace amount.^[^ ^125^ ^]^ Incorporating Y^3+^ in ZnSO_4_ electrolyte can also create an electrostatic shielding layer to regulate deposition behavior, leading to a high capacity retention of 89.6% after 2000 cycles at 5 A g^−1^.^[^ ^126^ ^]^

In addition to ionic additives, some organic materials can function as an electrostatic shielding layer. A study indicated that highly polarized diethyl ether molecules can preferentially adsorb on initially formed Zn growth tips under a highly localized electric field to hinder the further deposition of Zn^2+^ at these tips (Figure 8C).^[^ ^127^ ^]^ These ether molecules cause the deposition of Zn^2+^ to be shifted to the flat region, and the initial morphological fluctuation of planar Zn metal electrodes is diminished. Consequently, a Zn–MnO_2_ battery with diethyl ether as an electrolyte additive has high initial CE of 95.6% at 50 mA g^−1^, long‐term cycling stability, and high capacity retention of 97.7% at 5 A g^−1^.

Electrolyte additives based on electrostatic shielding mechanism can effectively inhibit dendrite growth by forcing Zn deposition in adjacent flat regions, mainly include inorganic cations and small polarized molecules. This kind of additives can adsorb on growth tips inducing homogeneous Zn deposition process because of the physically electrostatic field effect.

### 3.2 Adsorption

**Figure 9:**
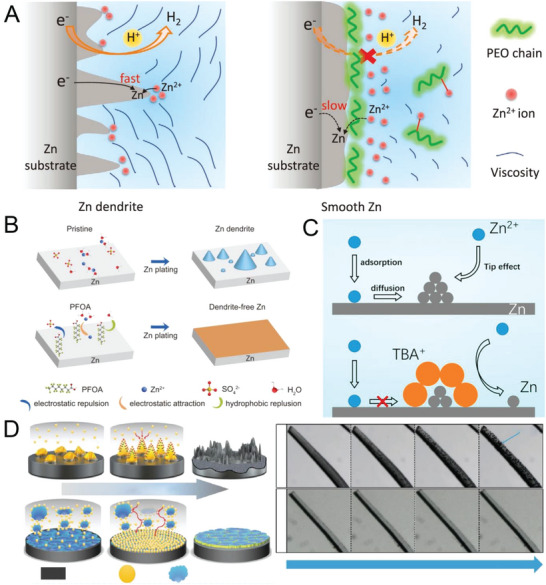
A) Schematic of electrochemical Zn deposition in pristine electrolyte and in electrolyte containing polyethylene oxide molecules as an additive. Reproduced with permission.^[^ ^128^ ^]^ Copyright 2020, John Wiely and Son. B) Schematic of ions in pristine electrolyte and in electrolyte with perfluorooctanoic acid as an additive. Reproduced with permission.^[^ ^129^ ^]^ Copyright 2022, Elsevier. C) Schematic of the Zn^2+^ diffusion and reduction processes on electrodes in 2 m ZnSO_4_ electrolyte and in 2 m ZnSO_4_ electrolyte containing 0.05 mm tetrabutylammonium sulfate as an additive. Reproduced with permission.^[^ ^130^ ^]^ Copyright 2020, American Chemical Society and D) schematic of the effect of an MXene additive on the Zn deposition process and in‐situ optical microscopy images of Zn deposition in a blank electrolyte and an MXene‐containing electrolyte. Reproduced with permission.^[^ ^133^ ^]^ Copyright 2021, Springer Nature.

Electrolyte additives adsorbed on a Zn anode can induce uniform plating that improves the life span of the anode by regulating the Zn^2+^ flux. On the basis of the adsorption principle, surfactant‐type electrolyte additives are commonly used in AZIBs. Jin et al. reported that long‐chain polyethylene oxide polymers can increase the cycle life and CE of a Zn anode when used as an electrolyte additive.^[^ ^128^ ^]^ Polyethylene oxide molecules adsorbed on Zn electrode interfaces ensure uniform anode surface chemistry and improve electrochemical stability, as shown in **Figure** **9**A. These electrochemically inert electrolyte additives can regulate the Zn^2+^ concentration distribution and electrolyte flux through interactions between their ether groups and Zn^2+^ and through electrolyte viscosity modification. Fluorinated anionic surfactant additives have been reported. For example, a perfluorooctanoic acid adsorption layer with strong electronegative perfluoroalkyl chains causes redistribution of the Zn^2+^ flux near the electrode–electrolyte interface and inhibits the migration of sulfate anions to the Zn anode (Figure 9B).^[^ ^129^ ^]^ This surfactant‐type electrolyte additive can also reduce the surface tension and improve the wettability of electrolytes on Zn electrode. The introduction of even a trace quantity of perfluorooctanoic acid electrolyte additive causes remarkable improvements in the reversibility of the Zn plating process and overall battery performance, with the specific capacity reaching 153 mAh g^−1^ at 5 A g^−1^ and excellent cycling stability being achieved with high CE at 2 A g^−1^. Tetrabutylammonium sulfate (TBA_2_SO_4_) was the first cationic surfactant‐type electrolyte additive to be reported. This additive induces uniform Zn deposition in the electrode preparation and battery charge–discharge processes (Figure 9C).^[^ ^130^ ^]^ Nonredox TBA^+^ ions are electrostatically adsorbed on the surface of the Zn electrodes; these ions regulate the initial nuclei formation and inhibit dendrite growth by providing shielding against hydrated Zn^2+^ in the electrolyte. Another cationic surfactant‐type electrolyte additive, cetyltrimethyl ammonium bromide can also adsorb onto the Zn anode to regulate Zn^2+^ deposition orientation and inhibit dendrite growth.^[^ ^131^ ^]^ In addition, an anionic surfactant known as sodium 3‐mercapto‐1‐propanesulfonate was reported to adsorb on the Zn anode due to the strong affinity of the sulfonate group and induce Zn deposition in the (002) direction, leading to outstanding capacity retention of 86.6% after 250 cycles in a 42 mAh pouch‐type Zn/V_2_O_5_·H_2_O full cell.^[^ ^132^ ^]^

Apart from surfactants, other materials can be used as electrolyte additives for enhancing the performance of Zn anodes through the adsorption mechanism. Some organic molecules, such as ethylene glycol and monomethyl ether, can chemisorb to the Zn surface and coordinate with Zn^2+^ to control the solvation structure, which moderately decreases the surface tension of aqueous electrolytes to increase the force driving Zn nucleation and growth, thereby promoting uniform deposition and preventing side reactions.^[^ ^146^ ^]^ In one study, a Ti_3_C_2_T*_x_* MXene with a large surface area, abundant surface functional groups, high metal conductivity, and strong hydrophilicity was used as an electrolyte additive to improve the irreversibility and kinetics of the Zn plating–stripping process.^[^ ^133^ ^]^ Zn^2+^ and MXene electrolyte additives combine because of electrostatic interaction and then adsorb on the surface of the Zn electrodes to form a conductive buffer layer. This MXene–Zn^2+^ adsorption layer can homogenize the distribution of surface Zn^2+^ and act as desired “seed points” for uniform nucleation, thereby inducing even Zn deposition without dendrite growth, as shown in Figure 9D. When MXene electrolyte additives are used, Zn anodes exhibit excellent cycling performance of more than 1100 cycles at 2 mA cm^−2^ and CE of nearly 100%.

Electrolyte additives based on an adsorption mechanism can produce a protecting layer on the surface of the Zn anode. The adsorption‐type additives conclude surfactants based on polymers, anions, cations, and others such as polymer molecules and MXenes. This kind of additive could effectively induce homogeneous plating and regulate the solvation sheath structure of zinc ions.

### 3.3 In Situ SEI Formation

In addition to creating an artificial electrode–electrolyte interface, many electrolyte additives can cause the in situ production of an SEI layer on Zn metal electrodes. In aqueous electrolytes, an SEI film is generated by the interfacial reactions between the electrodes and the electrolyte in the initial charging–discharging cycles. In these reactions, parts of the Zn^2+^ and electrolyte are consumed to form insoluble organic, inorganic, or organic–inorganic hybrid compounds, noted as SEI film. The formed SEI film can insulate the electrolyte from the Zn anode, thereby preventing the co‐embedding of solvent electrolyte molecules into the deposited Zn; thus, the Zn anode can be stabilized, and the life span of the AZIB can be increased.^[^ ^147^, ^148^, ^149^, ^150^ ^]^ However, producing a stable in situ SEI on a Zn anode in aqueous electrolyte remains challenging because of the relatively high reduction potential of Zn plating. Moreover, salt anions are difficult to decompose, and the decomposition products of water are detrimental gases rather than stable interphases. The ideal SEI layer for AZIBs must be robust against volume changes in cycling, have high Zn^2+^ conductivity, and be able to achieve self‐passivating growth.^[^ ^151^ ^]^ Various electrolyte additives have been investigated for enhancing the performance of Zn anodes through the in‐situ formation of an SEI.

**Figure 10:**
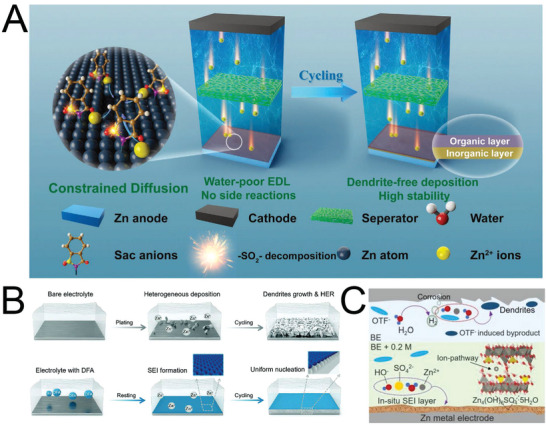
A) Schematic of Zn deposition in saccharin–ZnSO_4_ electrolyte and ZnSO_4_ electrolyte. Reproduced with permission.^[^ ^134^ ^]^ Copyright 2021, John Wiely and Son. B) Comparison of the Zn anode's stability in pure electrolyte (upper) and in electrolyte containing a compounding corrosion inhibitor (lower). Reproduced with permission.^[^ ^135^ ^]^ Copyright 2022, John Wiely and Son. C) Schematic illustration of the surface chemistry on Zn electrodes in baseline electrolyte (up) and baseline electrolyte + 0.2 m (down). Reproduced with permission.^[^ ^136^ ^]^ Copyright 2022, Elsevier.

The introduction of saccharin additive to aqueous ZnSO_4_ electrolyte can stabilize the electrode–electrolyte interface.^[^ ^134^ ^]^ Anions derived from saccharin that have higher adsorption energy than water molecules can form an electrical double layer near Zn electrodes before cycling; this layer then decomposes to form an SEI layer during cycling, as shown in **Figure** **10**A. The formed electrical double layer can inhibit side reactions by isolating water from Zn and prevent dendrite growth by modulating Zn^2+^ diffusion on the Zn surface. The SEI layer can also lead to uniform Zn deposition by inhibiting the random diffusion of Zn^2+^ on the surface of the Zn anode. Polymer electrolyte additives can also cause the in situ production of an SEI. For example, a compounding corrosion inhibitor composed of dodecyl dimethyl amine ethanolactone, fatty acid methyl ester ethoxylate, and fatty alcohol polyoxyethylene ether sodium sulfate was used as an additive for ZnSO_4_‐based electrolyte.^[^ ^135^ ^]^ When the aforementioned three units are combined, dimethyl amine ethanolactone acts as an electron donator, fatty acid methyl ester ethoxylate and fatty alcohol polyoxyethylene ether sodium sulfate act as electron acceptors, and the Zn surface serves as a platform for electron transfer; thus, a dense SEI layer with a thickness of ≈30 nm forms on Zn electrodes when using the aforementioned inhibitor (Figure 10B). This SEI layer can efficiently inhibit water corrosion and cause homogeneous Zn deposition without obvious dendrite growth, which enables reversible Zn anode cycling for over 1000 h at 1 mA cm^−2^ and 1 mAh cm^−2^ in Zn–Zn symmetric cells.

Inorganic electrolyte additives, such as SO_4_^2−^ additives, can also be used to produce a hydroxide‐based SEI layer for robust Zn anodes.^[^ ^136^ ^]^ An SEI layer composed of zinc hydroxide sulfate hydrate is produced by the self‐terminated chemical reactions of SO_4_^2−^ with Zn^2+^ and OH^−^, which are generated through the HER during the initial charging–discharging cycles (Figure 10C). The dense SEI layer uniformly coated on Zn can isolate active Zn from the electrolyte to terminate the continuous HER and corrosion. Furthermore, the SEI layer allows facile Zn^2+^ diffusion and homogenizes the plating behavior of Zn^2+^ to suppress dendrite growth. The resultant Zn electrode achieves high reversible performance with CE of 99.8% after 600 cycles and ultra‐long cycling stability of more than 2000 h in Zn–Zn symmetric cells.

Electrolyte additives based on in‐situ SEI formation include inorganic salts, organic molecules, and polymer esters, which can react with Zn anode, forming a dense SEI layer. These additives can effectively inhibit corrosions or side effects by isolating direct contact and guide the uniform Zn plating by regulating the uniform Zn^2+^ distribution.

### 3.4 Enhancing Water Stability

The chemical environments of aqueous electrolytes contain free water molecules and a primary solvation sheath.^[^ ^10^, ^152^, ^153^, ^154^ ^]^ The presence of a large quantity of free water with high chemical activity as well as free‐water molecules outside the Zn^2+^ solvation sheath results in the HER and corrosion of the Zn anode, which lead to poor reversibility.^[^ ^155^, ^156^ ^]^ The introduction of electrolyte additives can effectively modify the chemical environment of the electrolyte to enhance thermodynamic stability such that nonelectrolyte solutes can form hydrogen bonds to reduce the amount of free water and cosolvents and antisolvents can replace water molecules in the primary solvation sheath of Zn^2+^ cations.

**Figure 11:**
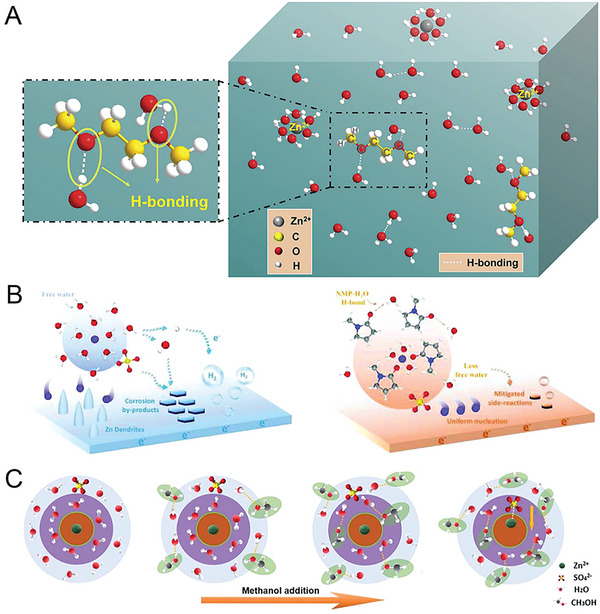
A) Schematic of the hydrogen bonding in an aqueous system with a low concentration of 1,2‐dimethoxyethane. Reproduced with permission.^[^ ^137^ ^]^ Copyright 2020, Elsevier. B) Schematic of the Zn^2+^ solvation structure and corresponding deposition behavior without and with *N*‐methyl‐2‐pyrrolidone electrolyte additive. Reproduced with permission.^[^ ^139^ ^]^ Copyright 2022, John Wiely and Son. C) Schematic of changes in the Zn^2+^ solvent sheath with methanol addition. Reproduced with permission.^[^ ^140^ ^]^ Copyright 2021, John Wiely and Son.

Similar to water‐in‐salt electrolytes, nonelectrolyte solute additives with abundant hydroxyl groups can break the tetrahedral (DDAA) bonded structure of free water and form hydrogen bonds with water molecules to suppress the reactivity of water and thus alleviate the problems caused by water. The versatile solvent 1,2‐dimethoxyethane can be used as an electrolyte additive to improve the performance of a Zn anode.^[^ ^137^ ^]^ 1,2‐Dimethoxyethane, which has unlimited mutual solubility in water, can form hydrogen bonds through the hydrophilic hydration of its ether oxygen and water molecules to alleviate the corrosion reactions induced by free water and impede chemical corrosion in mild acidic electrolyte (**Figure** **11**A). Other electrolyte additives with abundant hydroxyl groups can also bind with water molecules in aqueous electrolytes to strengthen O–H bonds for enhancing thermodynamic stability.^[^ ^154^, ^157^ ^]^ In a previous study, small molecules containing a dipole were used to develop aqueous electrolytes with high safety and a wide window of electrochemical stability.^[^ ^158^ ^]^ These molecules can break the network of hydrogen bonds between water molecules and considerably reduce the number of water molecules in the solvation sheath of the charge carrier.^[^ ^158^ ^]^ In one study, aqueous electrolytes with molecular crowding were produced using a water‐miscible polymer, namely poly(ethylene glycol), and then used to stabilize water molecules to obtain a wide voltage window and excellent HER stability; the water molecules were stabilized by being confined in a poly(ethylene glycol) network through hydrogen bonding.^[^ ^159^ ^]^

The solvation structure of the electrolyte can remarkably affect the chemistry at the electrode–electrolyte interface. In conventional aqueous electrolytes, Zn^2+^ is solvated by six water molecules in the primary solvation sheath; anions and the remaining unsolvated water molecules are excluded from the solvation sheath. Cosolvent additives with high Gutmann donor numbers can replace water molecules in the primary solvation sheath to coordinate with Zn^2+^ cations.^[^ ^160^, ^161^ ^]^ For instance, in one study, N,N‐dimethyl acetamide, which has a high Gutmann donor number of 27.8, was adopted to reconstruct the Zn^2+^ solvation sheath.^[^ ^138^ ^]^ *N*,*N*‐dimethyl acetamide can replace coordinated water molecules in the solvated Zn^2+^ sheath as well as confine free water and reconstruct the hydrogen bonding network in the aqueous electrolyte; thus, the introduction of this additive can produce an unexpectedly stable Zn plating–stripping process with an extended operating life span of 4500 h at 1 mA cm^−2^. Carbonyl‐containing organic polar solvents—such as *N*‐methyl‐2‐pyrrolidone, *N*,*N*‐dimethylformamide, and acetone—tend to preferentially solvate Zn^2+^ cations to reduce the quantity of free water outside the solvation sheath. Moreover, these cations serve as proton acceptors that bond with dipolar water molecules to destroy the original water clusters (Figure 11B).^[^ ^139^ ^]^ Through the aforementioned solvation modulation strategy, a reversible Zn plating–stripping process with CE of 99.7% can be achieved over 1000 cycles in Zn–Cu asymmetric cells.

Antisolvents are miscible with water, whereas salts are insoluble; thus, antisolvents can replace water molecules in primary solvation sheaths to promote cation–anion coordination for enhancing anode performance. Hao et al. reported a strategy in which methanol is used as an antisolvent to minimize water activity and weaken Zn^2+^ solvation.^[^ ^140^ ^]^ As the volume ratio of the additive increases, methanol molecules are gradually inserted into the outer and inner sheaths of Zn^2+^, which ultimately disrupts the coordination balance of water and Zn^2+^ in the inner sheath and leads to recrystallization (Figure 11C). Thus, with a suitable volume ratio of antisolvents, the water‐induced HER and corrosion reactions can be suppressed because of the diminished activity of free water and weakened Zn^2+^ solvation in the electrolyte.

Using electrolyte additives based on enhancing water stability mechanism has been widely adopted in AZIB research, which mainly contains nonelectrolyte solute with abundant hydroxyl groups and antisolvents that cannot dissolve salts. This kind of electrolyte additive can optimize the solvation sheath structure to inhibit HER and side reactions for high‐performance Zn anode.

### 3.5 Surface Texture Regulation

The crystallography and surface texture of Zn anodes have crucial influences on their performance in AZIBs. Texture, which refers to the preferred orientation of a Zn anode at the electrode–electrolyte interface, directly affects the direction of dendrite growth and the corrosion resistance. Among the major crystal planes of Zn—(100), (101), (002), (102), and (103)—the (100) plane is most prone to dendrite growth, whereas the (002) and (103) planes have the lowest surface energy, which leads to parallel growth along the Zn surface.^[^ ^162^, ^163^, ^164^, ^165^ ^]^ Accordingly, electrolyte additives can be used to modify the surface texture of Zn anodes with preferentially exposed flat and compact (002) planes for suppressing dendrite growth and the HER so that the anode reversibility is enhanced.

**Figure 12:**
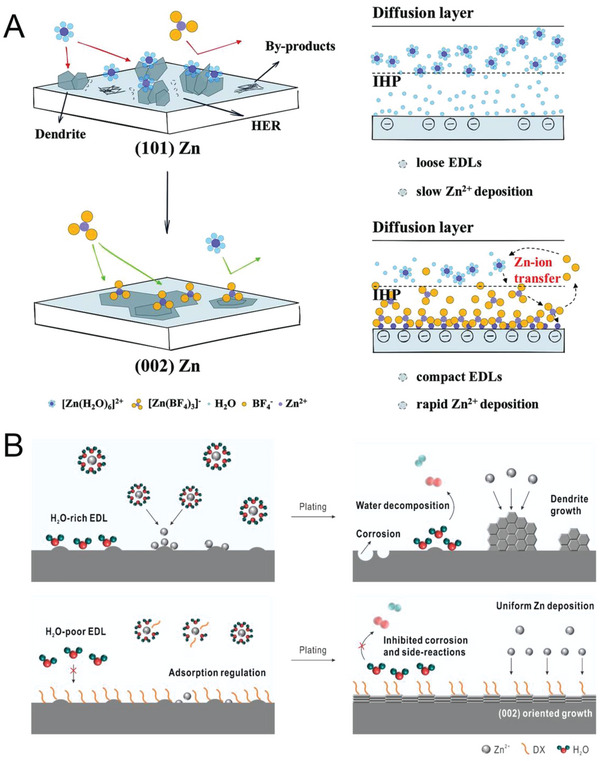
A) Schematic of (002)‐facet modulation and the mass transfer of Zn^2+^ when using Zn(BF_4_)_2_ additive. Reproduced with permission.^[^ ^141^ ^]^ Copyright 2022, John Wiely and Son and B) schematic of Zn deposition in bare ZnSO_4_ electrolyte and DX–ZnSO_4_ electrolyte. Reproduced with permission.^[^ ^143^ ^]^ Copyroght 2023, American Chemical Society.

Some inorganic and metal electrolyte additives can effectively regulate crystal planes. Zhu et al. reported a functional and low‐cost zinc tetrafluoroborate electrolyte additive that was found to lead to growth of the Zn(002) plane through the anionic function and the simultaneous involvement of Zn^2+^ transfer process, thereby enhancing the Zn anode's stability and the Zn plating–stripping efficiency.^[^ ^141^ ^]^ When zinc tetrafluoroborate is added to the electrolyte, BF_4_^−^ anions tend to replace water molecules in the Zn^2+^ solvation sheath to form the [Zn(H_2_O)_6−2_ *_x_* (BF_4_)*_x_*]^2−^ *^x^* complex because of the extremely electronegative F atoms in BF_4_^−^ anions. The [Zn (BF_4_)_3_]^−^ complex exhibits high adsorption energy for the (002) facet parallel to the close‐packed plane of Zn, which enhances the in‐plane growth and inhibits the upward growth of other planes, thereby leading to Zn(002) becoming the thermodynamically preferred facet for deposition (**Figure** **12**A). Other metal and inorganic additives—such as Pb, Sn,^[^ ^166^ ^]^ indium sulfate, tin oxide, and boric acid^[^ ^167^ ^]^—can also regulate surface texture to enhance the performance of Zn anodes.

Organic electrolyte additives can regulate surface texture and surface crystal structure to inhibit dendrite formation and enhance corrosion resistance. In one study, the roles of different organic additives—including cetyltrimethylammonium bromide, sodium dodecyl sulfate, polyethylene‐glycol, and thiourea—in the electroplating process were investigated.^[^ ^142^ ^]^ The results indicated that these organic additives can produce distinctive crystallographic orientations and surface textures. Sodium dodecyl sulfate is the most preferred of the aforementioned organic additives and effectively inhibits side reactions (float current, dendrite formation, and corrosion); additionally, it leads to 79% of a battery's capacity being retained after 1000 cycles. The addition of 1.4‐dioxane to electrolyte can regulate the nucleation and growth of Zn ions, thereby resulting in the promoted exposure of Zn(002) and the suppression of Zn dendrite formation (Figure 12B).^[^ ^143^ ^]^

Some inorganic, metal, and organic electrolyte additives can regulate surface texture and surface crystal structure through selective adsorption. These electrolyte additives promote the exposure of the Zn (002), leading to uniform Zn plating and suppressed side reactions through a surface texture regulation mechanism.

## 4 Summary and Future Outlook

Because of their low cost, satisfactory capacity, and high safety, AZIBs are promising batteries for grid EES systems. This review summarizes the major challenges involved in and current strategies used for enhancing the performance of Zn metal anodes in AZIBs, with particular emphasis on five functions of electrolyte additives, namely electrostatic shielding, adsorption, in situ SEI formation, enhancing water stability, and surface texture regulation. Although major achievements have been made in enhancing the life span and electrochemical performance of Zn electrodes through the use of different electrolyte additives, additional efforts must be made to achieve further progress in the aspects described in the following text.

First, the mechanism of electrode–electrolyte interface reactions, particularly when using functional electrolyte additives, should be further investigated. Research can be conducted on the solvated ion structure, the interactions between each component of the chemical system, and the evolution of interface structures through profound characterizations and calculations. The effective mechanism of electrolyte additives and its relation to electrolyte structures should be elaborated upon.

Second, novel electrolyte additives can be explored by considering the mechanisms of reaction and modification. The current information regarding electrolyte additives is still limited and mostly describes only single‐function additives. Thus, multifunction electrolyte additives that can enhance the performance of Zn anodes should be explored. Furthermore, electrolyte additives with differing effects on the functional requirements of different batteries can theoretically be designed in accordance with a profound understanding of the electrochemical reaction process.

Third, relevant studies have only used single electrolyte additives to improve the performance of Zn anodes. Therefore, future studies can use composite electrolyte additives for enhancing the performance of Zn anodes. The interactions between additives should be examined to determine their synergistic effects.

Fourth, the cost of the introduced electrolyte additives should be evaluated. Electrolyte additives are cheaper than are water‐in‐salt electrolytes. Cost‐efficient electrolyte additives with high performance can be developed in future studies.

Finally, considering cost and adjustability, anodes made of Zn powder are more suitable for commercial application than are anodes made of Zn foil; however, anodes made of Zn powder are more likely to corrode than are those made of Zn foil. Therefore, additional attention should be paid to electrolyte additives for anodes made of Zn powder.

More critically, the current literature data are mostly collected from button cells with a very high N/P ratio, a massive amount of electrolyte, and a very low areal capacity. By these configurations, it is easy to collect long cycling data, but the battery has an extremely low energy density. If the energy density is considered, that is, an appropriate N/P ratio, lean electrolyte, and high areal capacity, all these reported additives may not be that effective anymore. We suggest the community provides data collected from a more practical testing condition, such as soft pack batteries with an energy density of tens of Wh L^−1^ (although still low, much higher than that of button cells), even though they are not as attractive as data collected from button cells.

In fact, while we do believe in future commercialized rechargeable zinc ion batteries, electrolyte additives are certainly an important component; we also think a combination of other strategies to stabilize the Zn anode is essential. In current studies, researchers only adopt one method to stabilize the Zn anode for better scientific clarity. On the other hand, we believe multi‐additives for different functions, including HER inhibition, SEI, and cathode electrolyte interphase formation, will be adopted. Meanwhile, surface coating to the Zn anode will be needed for ion redistribution and dendrites suppression. We wish to have more studies on combined strategies targeting stable Zn anode and more data collected on critical testing conditions to lead the community's healthy development.

## Conflict of Interest

The authors declare no conflict of interest.
